# Estimates of meteorological variability in association with dengue cases in a coastal city in northern Vietnam: an ecological study

**DOI:** 10.3402/gha.v7.23119

**Published:** 2014-12-08

**Authors:** Le Thi Thanh Xuan, Pham Van Hau, Do Thi Thu, Do Thi Thanh Toan

**Affiliations:** 1 Department of Environmental Health, Institute for Preventive Medicine and Public Health, Hanoi Medical University, Hanoi, Vietnam; 2 Pasteur Institute in Ho Chi Minh City, Ho Chi Minh City, Vietnam; 3 Department of Biostatistics and Medical Informatics, Institute for Preventive Medicine and Public Health, Hanoi Medical University, Hanoi, Vietnam

**Keywords:** dengue fever, coastal city, Vietnam, weather, meteorology

## Abstract

**Background:**

Dengue fever (DF) is a vector-borne disease that is sensitive to weather and climate variability. To date, however, this relationship in coastal northern Vietnam has not been well documented.

**Objectives:**

This paper aims to examine the associations between meteorological variables and dengue incidence in Haiphong, Vietnam, over the period 2008–2012.

**Methods:**

Monthly data on dengue incidence from all commune health stations and hospitals of Haiphong (with a total population of ~1.8 million) were obtained in accordance with the WHO's recommendations over a 5-year period (2008–2012). Temperature, rainfall, and humidity were recorded as monthly averages by local meteorological stations. The association between ecologic weather variables and dengue cases was assessed using a Poisson regression model. The estimation of regression parameters was based on the method of maximum likelihood using the R program package.

**Results:**

From 2008 through 2012, 507 cases of dengue were reported. The risk of dengue was increased by sevenfold during the September–December period compared with other months over the period 2008–2012. DF cases in Haiphong were correlated with rainfall and humidity. In the multivariable Poisson regression model, an increased risk of dengue was independently associated with months with a higher amount of rainfall (RR=1.06; 95% CI 1.00–1.13 per 50 mm increase) and higher humidity (RR=1.05; 95% CI 1.02–1.08 per 1% increase).

**Conclusion:**

These data suggest that rainfall and relative humidity could be used as ecological indicators of dengue risk in Haiphong. Intensified surveillance and disease control during periods with high rainfall and humidity are recommended. This study may provide baseline information for identifying potential long-term effects and adaptation needs of global climate change on dengue in the coming decades.

Dengue fever (DF), a climate-sensitive mosquito-transmitted viral infection, is a public health problem on the rise in many tropical regions (1–6). According to the World Health Organization (WHO), DF has re-emerged globally and has increased over space and time since the 1980s. The disease is currently endemic to more than 100 countries with an estimated approximately 100 million people infected every year worldwide (7). DF outbreaks were recorded as early as the late 1940s in South East Asia (8), and the disease has become a major public health burden in the region for several decades (9, 10). If global climate continues to change, it is estimated that about 50–60% of the projected global population would be at risk of dengue transmission (11). In addition to the health consequences, the disease also creates an economic burden at family and societal levels (12).

Some weather types and climate fluctuations have been shown to contribute to increasing DF incidence (7, 13–24). A few previous studies have indicated an association between specific meteorological variables and dengue incidence using time series analyses. They found that rainfall (3, 18, 22, 25, 26), high relative humidity (3, 14, 26), and high temperature (7, 10, 16, 20, 23, 27–30) correlated with DF through increased survival time and shortening of the development cycle of mosquitoes that subsequently increased the possibility of transmission of DF among other mechanisms. The strongest weather–dengue case correlation was associated with combining the three variables: rainfall, humidity, and high temperature (22, 24, 31). Identification of the association between DF and weather could, at present, serve as a basis for early warning systems, surveillance, and prediction, and in the longer run help adapt to the potential negative impacts that climate change can have on dengue disease proliferation (10, 22, 28, 30, 32). In addition, it will be also useful for effective prevention and control strategies in regions with limited resources, such as developing countries (29, 30).

Vietnam's climate is humid and tropical, with seasonal and geographical variance (33, 34). Overall, the climate is generally favorable for mosquitoes to transmit DF. DF was first described in Vietnam in 1958 (35) and the first reported outbreak occurred in southern Vietnam in 1963 (36). Dengue transmission occurs throughout the year in Vietnam, with peak numbers of cases (72% of total cases) reported between June and November. However, there are regional differences in the climate in Vietnam and therefore seasonal transmission patterns can vary. The Northern and Central Highland regions have a cool, dry winter from December to March each year, and dengue notifications are low during this time, whereas in southern Vietnam that has a warm stable climate throughout the year, the peak transmission occurs between July and September, coinciding with the rainy season (37).

Previous studies found that high temperature and rainfall were associated with the increase of dengue case in Vietnam (1, 12, 37–42). However, the association between weather and dengue and, subsequently, climate changes and DF in a coastal city in northern Vietnam is currently not well understood (42). The current paper aims to examine the relationship between weather factors and DF incidence among the population in Haiphong, a coastal city in northern Vietnam, over the period 2008–2012. The findings of this study could result in better control planning, surveillance, and prevention strategies to date in the region, and to the development of adaptation strategies to minimize potential negative impacts of climate change on dengue incidence.

## Methods

### Study settings

**Fig. 1:**
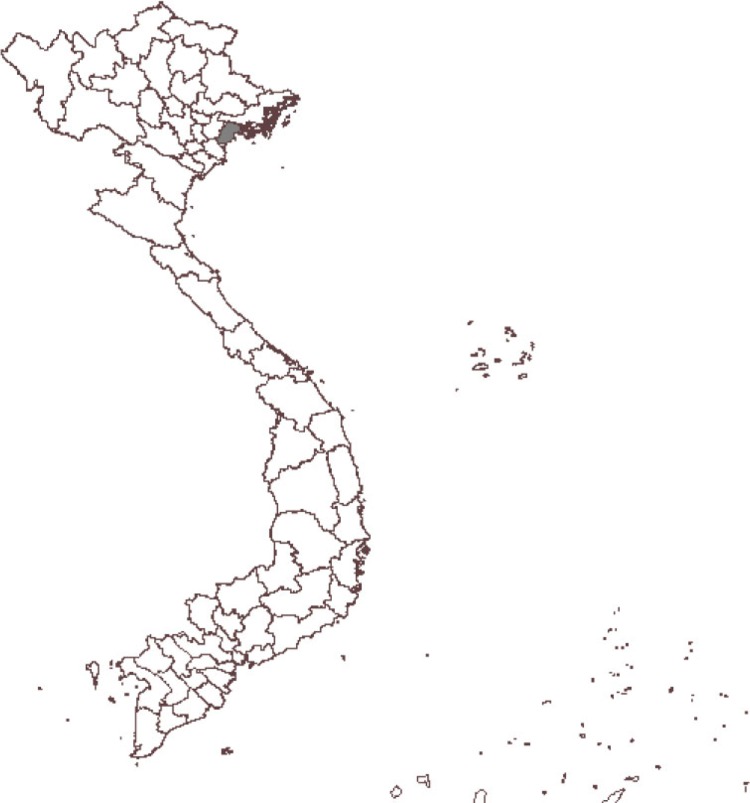
Map of Vietnam showing Haiphong province (in dark grey area).

The study was conducted in Haiphong (Fig. 1), with the total square of 152.300 ha. The administrative levels in Vietnam are nation, province, district, and commune, and there are five biggest provinces in Vietnam also named city. Haiphong city has a population of about 1.837 million people in 2009 (43). Haiphong's climate is typically hot, humid, and rainy. The summer period from May to September receives highest amount of rainfall in the year (1,600–1,800 mm rainfall/year). The average temperature during the year was 23–26°C with the highest figure in June and July. The annual average humidity was about 80–85% with levels peaking in July, August, and September and lowest in December and January.

### Data collection

In Haiphong, there is a network of health stations located in each commune. Each commune health station is in charge of dengue surveillance and reports the monthly surveillance data with dengue case notifications to the district level and later to Haiphong city's Center for Preventive Health. This surveillance system was mainly carried out by government staff, not by private practitioners.

Meteorological data, including temperature (°C), amount of rainfall (mm), and relative humidity (%), were obtained from the local bureaus of meteorology across Haiphong. Meteorological data in Haiphong comes from nine meteorological stations and 23 rain gauge stations distributed around this province. Data from these stations were aggregated to district, then city. The data represent monthly averages for each year during the study period 2008–2012.

### Surveillance and data analysis

The main aim of data analysis was to describe the dengue incidence and its association with weather variables. The outcome considered in the analysis was the actual number of dengue cases of the whole city, while the weather variables were monthly local (city and district), temperature, rainfall, and humidity. Because the number of dengue cases was small relative to the provincial population, it is reasonable to assume that the distribution of dengue cases followed the Poisson distribution. Accordingly, a zero-inflated Poisson regression model was used to model the relationships between the potential risk factors and dengue cases (44). Zero-inflated Poisson regression is a model for count data (number of cases) with excess zeros (districts had no cases in months), it assumes that with probability *p*, the only possible observation is 0, and with probability 1 – *p*, a Poisson (λ) random variable is observed. In this model, the log incidence of dengue, log(Y), was assumed to be related to a risk factor *X* by an additive linear function as follows: Log(Y)=*a*+*bX*+*e*, where *a* and *b* are regression parameters to be estimated from the observed data, and *e* represents the residual not explained by the variable *X*. As temperature, rainfall and humidity could be varied by month and locality, the relationship between these variables and dengue disease counts were adjusted for population, time (month, season, year), and area (urban, rural or island districts). Population was put in the model as an offset (log). The estimation of regression parameters was based on the method of maximum likelihood with the R program package (45). The fitted data from an optimal model was evaluated to the observed dengue disease counts by correlation and mean square error. Non-linear relationships such as polynomial regression analysis and square for satisfying normal distribution and stabilizing variance were considered but finally a model was chosen based on Akaike information criterion (AIC) (Table 3). Different types of functions, including linear, non-linear relationship, or data transformation, were used to examine factors related to dengue disease counts, and the best fit model was selected based on AIC; the model with the smaller AIC has the better fit.

## Results

**Fig. 2:**
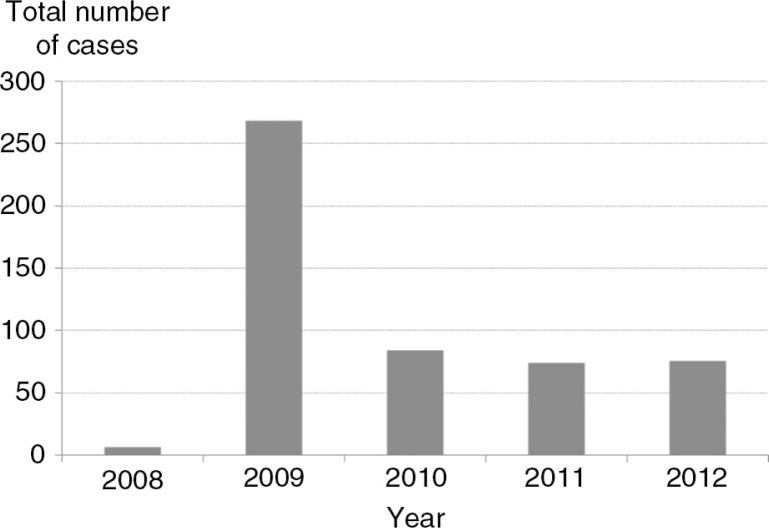
Total number of reported dengue fever cases in Haiphong, 2008–2012.

During the follow-up period (2008–2012), there were 507 cases of dengue reported in Haiphong, making the morbidity rate of 27.2 per 100,000 persons. The number of cases strongly fluctuated from year to year (Fig. 2) without any apparent systematic trend, and between months within a year. There was an epidemic in 2009 that accounted for 51.7% of cases during the surveillance period (Fig. 2).

**Fig. 3:**
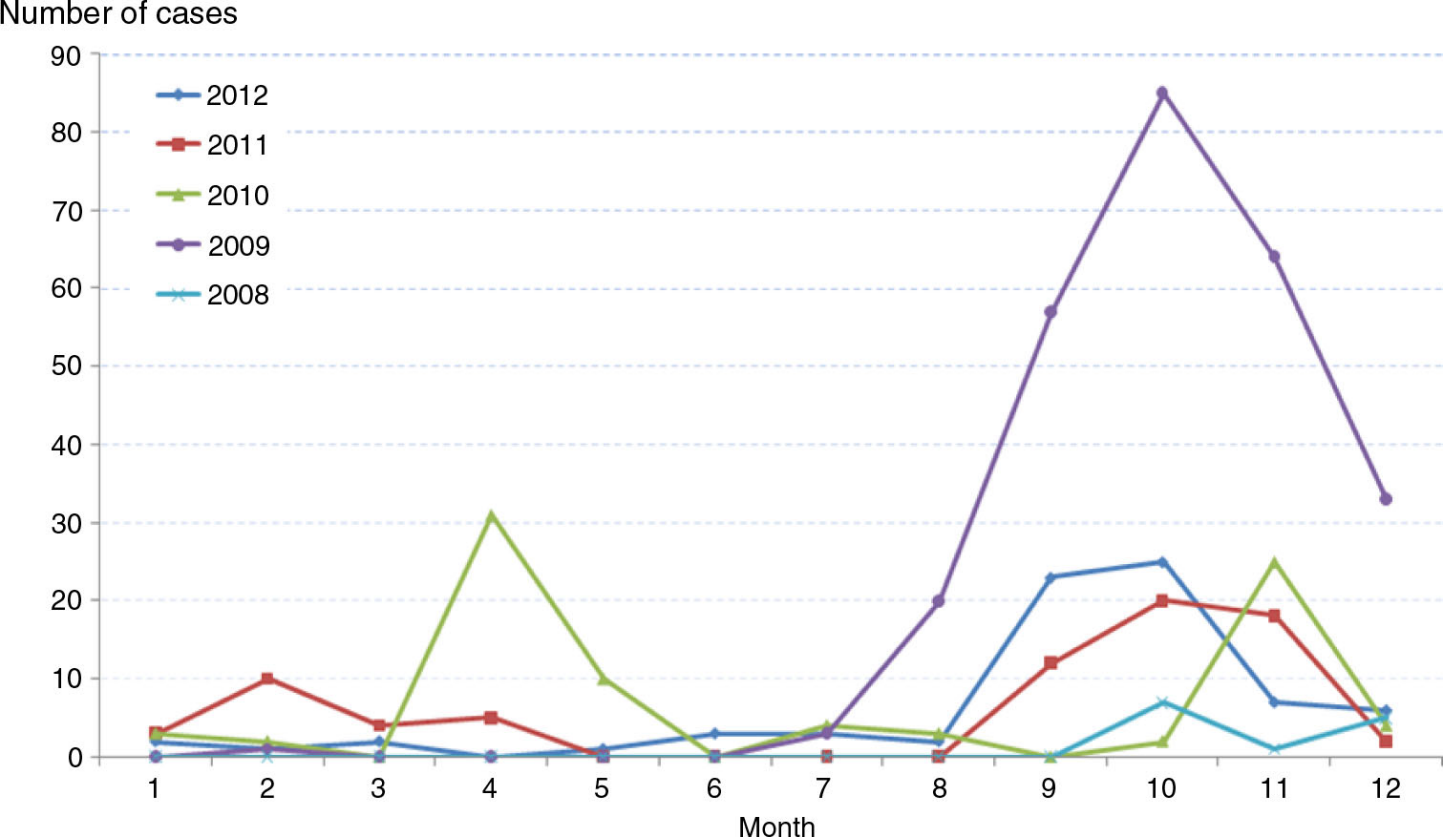
Number of reported dengue fever cases in Haiphong by month, 2008–2012.

Within a year, the number of dengue cases by month during the 2008–2012 period is shown in Table 1 and Fig. 3. The number of cases reported with a peak in the September–December period; from 2008 to 2012, the total number of dengue cases recorded during the September–December period accounted for 78% of total cases. On the other hand, the risk of dengue was increased by sevenfold during the September–December period compared with the other month.

**Table 1:** Meteorological factors and occurrence of dengue cases stratified by month

Month	No. of cases^a^	Temperature^b^ (°C)±SD	Rainfall^b^ (mm)±SD	Humidity^b^ (%)±SD
Jan	8	15.0±1.5	38.5±31.9	86.2±5.1
Feb	14	17.1±2.8	19.3±9.4	91.0±2.8
Mar	6	19.3±1.8	45.2±29.8	91.0±1.3
Apr	36	23.1±0.8	95.1±56.4	91.4±2.1
May	11	26.4±1.03	217.5±117.1	89.2±1.2
Jun	3	28.6±0.8	202.1±85.0	87.0±3.4
Jul	10	28.7±0.5	209.4±51.3	87.4±1.5
Aug	25	27.9±0.6	358.4±149.0	90.2±2.1
Sep	92	27.1±0.5	296.9±80.5	88.8±2.5
Oct	135	25.3±1.1	83.1±54.2	84.6±3.5
Nov	115	22.0±1.2	37.2±22.1	80.4±5.4
Dec	52	18.5±1.0	21.6±10.8	82.8±2.8
Total	507	23.2±4.8	137±133	87.5±4.5

a Data are total number of cases tallied from 2008 to 2012.

b Data are averages of 5 years (2008–2012).

Results of univariate analysis (Table 2) showed that after adjusting for seasonality, the risk of dengue was significantly associated with increased rainfall and increased humidity. The risk ratio was from 1.02 to 1.03 (*p*<0.05).

**Table 2:** Risk factors RR (95%CI) for dengue incidence in Haiphong: univariate analysis

Risk factors	Unit of comparison	RR (95%CI)	*p*
Temperature	Per 1°C increase	1.02 (0.99–1.05)	>0.05
Rainfall	Per 50 mm increase	1.03 (1.00–1.07)	<0.05
Humidity	Per 1% increase	1.02 (1.00–1.05)	<0.05

**Fig. 4:**
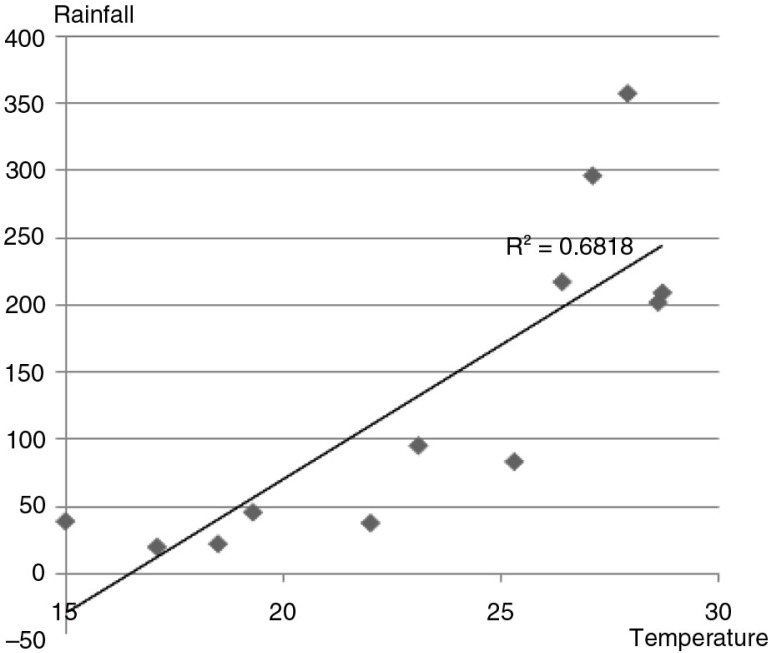
Scatter plot of monthly mean temperature and rainfall.

There was a significant correlation between monthly mean temperature and rainfall with *r*=0.68; *p*<0.00001 (Fig. 4); a multivariable Poisson regression model was fitted to the data to search for independent factors.

In the multivariable Poisson regression model (Table 3), an increased risk of dengue was independently associated with months with a higher amount of rainfall (RR=1.06; 95% CI 1.00–1.13 per 50 mm increase) and higher humidity (RR=1.05; 95% CI 1.02–1.08 per 1% increase). The final Poisson regression model (Table 3) with two meteorological variables showed 11.7% climate factors contributing to the occurrence of dengue cases.

**Table 3:** Risk factors RR (95%CI) for dengue incidence in Haiphong: multivariate analysis

Risk factors	Unit of comparison	RR (95%CI)	*p*
Mean temperature	Per 1°C increase	0.97 (0.94–1.01)	>0.10
Rainfall	Per 50 mm increase	1.06 (1.00–1.13)	<0.05
Humidity	Per 1% increase	1.05 (1.02–1.08)	<0.001

## Discussion

A previous study found that climate factors had positive effects in increasing DF transmission in Vietnam (39–42). Particularly, rainfall and relative humidity affected the rates of biological development, feeding, reproduction, population density, and survival of Aedes mosquitoes, thus increasing the DF transmission. The present study illustrated DF reported in a coastal city in northern Vietnam with the highest number of cases from September to December over the period 2008–2012. Rainfall and relative humidity were correlated with an increased occurrence of DF, while elevated temperature was not associated in this study.

Seasonality in the incidence of DF has been seen in a number of countries that can be explained by seasonal variation of climate factors. A study in Singapore reported that dengue incidence was generally higher during the June–October period (30). A study in Trinidad in the period 2002–2004 illustrated a dengue season between June and November (25). In Vietnam, previous studies also demonstrated that most cases were reported during the rainy season, July–December (1, 39, 41). However, the number of DF cases in the present study does not increase at the beginning of the high-rainfall and relative humidity in Haiphong. It was observed that the rainfall (358.4 mm) and relative humidity (90.2%) reached the highest level in August then started decreasing in September, when the number of DF cases started increasing. Previous studies have reported that dengue epidemics take several months to reach a level where they can be recognized as the result of previous climate conditions (46, 47). This finding provides data for further investigation of ‘delay effects’ of rainfall and relative humidity for better prediction, planning, and intervention.

Our result is consistent with previous studies, which found that rainfall and humidity were associated with the number of DF incidence in developing countries (3, 14, 18, 22, 25, 26). Rainfall and relative humidity are found to increase survival time and shorten the development cycle of mosquitoes (3), resulting in an increased number of breeding sites and adult female mosquitoes (7, 13–24). However, heavy rainfall may also reduce the survival rate of adult mosquitoes. An increase in the number of mosquitoes increases the probability of DF transmission (16). Our results implied that DF-control programs and surveillance need to be intensified during the period with high rainfall and high relative humidity, even when there is no apparent increase in the cases of DF.

In this study, the correlation between temperature and DF incidence was not significant. This result is similar to a study in Trinidad 2007 (25) and Metro Manila 1996–2005 (18) and in the Philippines (48). However, previous studies found that temperature was associated with the occurrence of DF (7, 10, 16, 20, 23, 27–30). Particularly, the variable temperature (maximum and minimum) was the best predictor for the increased number of dengue cases in Singapore (23). However, in this study, the maximum and minimum temperatures for data collection were not available with recommendations for future analysis. Previous studies in Vietnam also found the temperature–dengue relationship in Hanoi (41) and Central Highlands (39). The difference in our study might be explained by the fact that the range of mean temperature in Haiphong was lower than other areas. In fact, being a coastal area, the temperature in Haiphong is often 1°C higher in winter and 1°C lower in summer compared to Hanoi capital (43). Furthermore, a study has shown that dengue viruses may reduce incubation time in mosquitoes from approximately 2 to 1 week at temperatures of 32°C and above (49), while the maximum of monthly mean temperature in our study was 29°C. It suggested that daily temperature data is needed if we want to measure the temperature–dengue relationship in a coastal area.

This study should be interpreted within the context of some strengths and potential limitations. The strength of this study is that it could ascertain most (if not all) dengue cases in Haiphong based on available public surveillance system. The health workers at all levels in the city are very familiar with DF identification and reporting systems. As a result, the low case-fatality rate and the standard clinical case definition for dengue cases in the whole city have been used for more than 10 years without any substantial change (6). However, the study used monthly data for DF cases, which might not be the ideal compared to daily counts (29). In addition, data based on surveillance systems might be underestimated. To our knowledge, there were a number of dengue patients treated in private health services that could not be recorded. A study of the diagnosis of acute undifferentiated fever in Vietnam showed that acute dengue was found in ~34% cases, which suggests the possibility of underreporting of dengue in commune health stations (50). WHO classification schemes in 1997 and 2009 had high sensitivity but lacked specificity (51). This study has demonstrated the effects of climate factors on the occurrence of DF but has not considered socio-economic, immunological determinants that have contributed to dengue (4, 52) and some other factors such as degree of urbanization (53), El Niño (19, 22), and mosquitoes index (31). The study to measure the association between DF and other social–economic variables needs to be investigated further.

## Conclusion

In summary, this study measured the correlation of meteorological factors–DF in Haiphong. Rainfall and relative humidity could be used as ecological indicators of dengue risk in Haiphong. Intensified control programs and surveillance during rainfall and high relative humidity seasons are necessary preventive measures. This study may provide baseline information for identifying the potential long-term effects of global climate change on dengue expected in the coming decades.

## References

[1] Cuong HQ, Vu NT, Cazelles B, Boni MF, Thai KT, Rabaa MA (2013). Spatiotemporal dynamics of dengue epidemics, southern Vietnam. Emerg Infect Dis.

[2] Guzman A, Isturiz RE (2010). Update on the global spread of dengue. Int J Antimicrob Agents.

[3] Nitatpattana N, Singhasivanon P, Kiyoshi H, Andrianasolo H, Yoksan S, Gonzalez JP (2007). Potential association of dengue hemorrhagic fever incidence and remote senses land surface temperature, Thailand, 1998. Southeast Asian J Trop Med Public Health.

[4] Thai KT, Anders KL (2011). The role of climate variability and change in the transmission dynamics and geographic distribution of dengue. Exp Biol Med (Maywood).

[5] WHO (2012). Dengue and dengue haemorrhagic fever. http://www.who.int/mediacentre/factsheets/fs117/en/.

[6] WHO/TDR (2009). Dengue: guidelines for diagnosis, treatment, prevention and control.

[7] Chowell G, Sanchez F (2006). Climate-based descriptive models of dengue fever: the 2002 epidemic in Colima, Mexico. J Environ Health.

[8] Gubler DJ (1998). Dengue and dengue hemorrhagic fever. Clin Microbiol Rev.

[9] Ooi EE, Gubler DJ (2009). Dengue in Southeast Asia: epidemiological characteristics and strategic challenges in disease prevention. Cad Saude Publica.

[10] Hii YL, Rocklov J, Ng N, Tang CS, Pang FY, Sauerborn R (2009). Climate variability and increase in intensity and magnitude of dengue incidence in Singapore. Glob Health Action.

[11] Hales S, de Wet N, Maindonald J, Woodward A (2002). Potential effect of population and climate changes on global distribution of dengue fever: an empirical model. Lancet.

[12] Harving ML, Ronsholt FF (2007). The economic impact of dengue hemorrhagic fever on family level in Southern Vietnam. Dan Med Bull.

[13] Fuller DO, Troyo A, Beier JC (2009). El Nino Southern Oscillation and vegetation dynamics as predictors of dengue fever cases in Costa Rica. Environ Res Lett.

[14] Halide H, Ridd P (2008). A predictive model for dengue hemorrhagic fever epidemics. Int J Environ Health Res.

[15] Jetten TH, Focks DA (1997). Potential changes in the distribution of dengue transmission under climate warming. Am J Trop Med Hyg.

[16] Johansson MA, Dominici F, Glass GE (2009). Local and global effects of climate on dengue transmission in Puerto Rico. PLoS Negl Top Dis.

[17] Promprou S, Jaroensutasinee M, Jaroensutasinee K (2005). Climatic factors affecting dengue haemorrhagic fever incidence in Southern Thailand. Dengue Bull.

[18] Su GLS (2008). Correlation of climatic factors and dengue incidence in Metro Manila, Philippines. AMBIO.

[19] Cazelles B, Chavez M, McMichael AJ, Hales S (2005). Nonstationary influence of El Niño on the synchronous dengue epidemics in Thailand. PLoS Med.

[20] Descloux E, Mangeas M, Menkes CE, Lengaigne M, Leroy A, Tehei T (2012). Climate-based models for understanding and forecasting dengue epidemics. PLoS Negl Trop Dis.

[21] Githeko AK, Lindsay SW, Confalonieri UE, Patz JA (2000). Climate change and vector-borne diseases: a regional analysis. Bull World Health Organ..

[22] Zambrano LI, Sevilla C, Reyes-Garcia SZ, Sierra M, Kafati R, Rodriguez-Morales AJ (2012). Potential impacts of climate variability on dengue hemorrhagic fever in Honduras, 2010. Trop Biomed.

[23] Pinto E, Coelho M, Oliver L, Massad E (2011). The influence of climate variables on dengue in Singapore. Int J Environ Health Res.

[24] Cassab A, Morales V, Mattar S (2011). [Climatic factors and cases of dengue in Monteria, Colombia: 2003–2008]. Rev Salud Publica (Bogota).

[25] Chadee DD, Shivnauth B, Rawlins SC, Chen AA (2007). Climate, mosquito indices and the epidemiology of dengue fever in Trinidad (2002–2004). Ann Trop Med Parasitol.

[26] Wiwanitkit V (2006). An observation on correlation between rainfall and the prevalence of clinical cases of dengue in Thailand. J Vector Borne Dis.

[27] Chen SC, Hsieh MH (2012). Modeling the transmission dynamics of dengue fever: implications of temperature effects. Sci Total Environ.

[28] Colon-Gonzalez FJ, Lake IR, Bentham G (2011). Climate variability and dengue fever in warm and humid Mexico. Am J Trop Med Hyg.

[29] Hii YL, Rocklov J, Wall S, Ng LC, Tang CS, Ng N (2012). Optimal lead time for dengue forecast. PLoS Negl Trop Dis.

[30] Hii YL, Zhu H, Ng N, Ng LC, Rocklov J (2012). Forecast of dengue incidence using temperature and rainfall. PLoS Negl Trop Dis.

[31] Yi B, Zhang Z, Xu D, Xi Y (2003). [Relationship of dengue fever epidemic to Aedes density changed by climate factors in Guangdong Province]. Wei sheng yan jiu [J Hyg Res].

[32] Dibo MR, Chierotti AP, Ferrari MS, Mendonca AL, Chiaravalloti Neto F (2008). Study of the relationship between *Aedes* (Stegomyia) *aegypti* egg and adult densities, dengue fever and climate in Mirassol, state of Sao Paulo, Brazil. Memorias do Instituto Oswaldo Cruz.

[33] United Nations (2011). Climate change Fact Sheet: the effects of climate change in Viet Nam and the UN's Responses. http://www.un.org.vn/en/publications/cat_view/130-un-viet-nam-joint-publications/209-climate-change-joint-un-publications.html.

[34] Ministry of Natural Resources and Environment (2009). Climate change, sear level rise scenarios for Vietnam.

[35] Mihov C, Tuong CV, Tuong HP (1959). A propos d une epidemie du type des fievres hemorragiques a Hanoi. Focia Medica.

[36] Ha DQ, Huan TQ (1997). Dengue activity in Vietnam and its control program, 1997–1998. Dengue Bull.

[37] Ha DQ, Tien NT, Huong VT, Loan HT, Thang CM (2000). Dengue epidemic in southern Vietnam, 1998. Emerg Infect Dis.

[38] Cuong HQ, Hien NT, Duong TN, Phong TV, Cam NN, Farrar J (2011). Quantifying the emergence of dengue in Hanoi, Vietnam: 1998–2009. PLoS Negl Trop Dis.

[39] Pham HV, Doan HT, Phan TT, Minh NN (2011). Ecological factors associated with dengue fever in a Central Highlands province, Vietnam. BMC Infect Dis.

[40] Thai KT, Cazelles B, Nguyen NV, Vo LT, Boni MF, Farrar J (2010). Dengue dynamics in Binh Thuan province, southern Vietnam: periodicity, synchronicity and climate variability. PLoS Negl Trop Dis.

[41] Toan do TT, Hu W, Quang Thai P, Hoat LN, Wright P, Martens P (2013). Hot spot detection and spatio-temporal dispersion of dengue fever in Hanoi, Vietnam. Glob Health Action.

[42] Tsuzuki A, Vu TD, Higa Y, Nguyen TY, Takagi M (2009). High potential risk of dengue transmission during the hot-dry season in Nha Trang City, Vietnam. Acta Trop.

[43] GSO (2009). Census of population and housing in Vietnam in 2009.

[44] Yang Y, Kang J, Mao K, Zhang J (2007). Regression models for mixed Poisson and continuous longitudinal data. Stat Med.

[45] R D Core Team (2005). R: A language and environment for statistical computing.

[46] Guzman MG, Kouri G, Valdes L, Bravo J, Alvarez M, Vazques S (2000). Epidemiologic studies on Dengue in Santiago de Cuba, 1997. Am J Epidemiol.

[47] Newton EA, Reiter P (1992). A model of the transmission of dengue fever with an evaluation of the impact of ultra–low volume (ULV) insecticide applications on dengue epidemics. Am J Trop Med Hyg.

[48] Su GL (2008). Correlation of climatic factors and dengue incidence in Metro Manila, Philippines. Ambio.

[49] Watts DM, Burke DS, Harrison BA, Whitmire RE, Nisalak A (1987). Effect of temperature on the vector efficiency of *Aedes aegypti* for dengue 2 virus. Am J Trop Med Hyg.

[50] Phuong HL, de Vries PJ, Nga TT, Giao PT, Hung le Q, Binh TQ (2006). Dengue as a cause of acute undifferentiated fever in Vietnam. BMC Infect Dis.

[51] Chaterji S, Allen JC, Chow A, Leo YS, Ooi EE (2011). Evaluation of the NS1 rapid test and the WHO dengue classification schemes for use as bedside diagnosis of acute dengue fever in adults. Am J Trop Med Hyg.

[52] Astrom C, Rocklov J, Hales S, Beguin A, Louis V, Sauerborn R (2012). Potential distribution of dengue fever under scenarios of climate change and economic development. EcoHealth.

[53] Wu PC, Lay JG, Guo HR, Lin CY, Lung SC, Su HJ (2009). Higher temperature and urbanization affect the spatial patterns of dengue fever transmission in subtropical Taiwan. Sci Total Environ.

